# Mechanistic Insights into the Antilithiatic Proteins from *Terminalia arjuna*: A Proteomic Approach in Urolithiasis

**DOI:** 10.1371/journal.pone.0162600

**Published:** 2016-09-20

**Authors:** Amisha Mittal, Simran Tandon, Surender Kumar Singla, Chanderdeep Tandon

**Affiliations:** 1 Department of Biotechnology and Bioinformatics, Jaypee University of Information Technology, Waknaghat, Solan, Himachal Pradesh, India; 2 Amity Institute of Molecular Medicine and Stem Cell Research (AIMMSCR), Amity University Uttar Pradesh, Noida, Uttar Pradesh, India; 3 Department of Biochemistry, Panjab University, Chandigarh, India; 4 Amity Institute of Biotechnology, Amity University Uttar Pradesh, Noida, Uttar Pradesh, India; University of Louisville, UNITED STATES

## Abstract

Kidney stone formation during hyperoxaluric condition is inherently dependent on the interaction between renal epithelial cells and calcium oxalate (CaOx) crystals. Although modern medicine has progressed in terms of removal of these stones, recurrence and persistent side effects restricts their use. Strategies involving plant based agents which could be used as adjunct therapy is an area which needs to be explored. Plant proteins having antilithiatic activity is a hitherto unexplored area and therefore, we conducted a detailed identification and characterization of antilithiatic proteins from *Terminalia arjuna* (*T*. *arjuna*). Proteins were isolated from the dried bark of *T*. *arjuna* and those having molecular weights > 3 kDa were subjected to anion exchange chromatography followed by gel filtration chromatography. Four proteins were identified exhibiting inhibitory activity against CaOx crystallization and crystal growth kinetics The cytoprotective and anti-apoptotic efficacy of these purified proteins was further investigated on oxalate injured renal epithelial cells (MDCK and NRK-52E) wherein, injury due to oxalate was significantly attenuated and led to a dose dependent increase in viability of these cells. These proteins also prevented the interaction of the CaOx crystals to the cell surface and reduced the number of apoptotic cells. Identification of these 4 anionic proteins from the bark of *T*. *arjuna* was carried out by Matrix-assisted laser desorption/ionization-time of flight Mass spectrometry (MALDI-TOF MS). This was followed by database search with the MASCOT server and sequence similarity was found with Nuclear pore anchor, DEAD Box ATP-dependent RNA helicase 45, Lon protease homolog 1 and Heat shock protein 90–3. These novel proteins isolated from *T*. *arjuna* have the potential to inhibit CaOx crystallization and promote cell survival and therefore, offer novel avenues which need to be explored further for the medical management of urolithiasis.

## Introduction

The urinary tract is prone to a number of adverse conditions which impact its functioning. One prominent urinary tract disease is urolithiasis, which is the third most frequent urological affliction in humans [1]. Stone disease affects 2–20% population worldwide [2] with a prevalence rate of 15% in India [3]. The occurrence of CaOx kidney stone is the net effect of a panoply of factors which manifests themselves both within the cells as well as the environment in which the cells are present. The composition of the urinary fluid in terms of various ions, the propensity of these ions to form crystals and their further growth, as well as the presence of macromolecules in the fluid, are some examples of extracellular factors which occur in the tubular lumen and pelvis of the kidney. Cellular events/triggers which take place in the renal epithelial and interstitial cells, comprise of the interaction of oxalate and/or CaOx crystals on renal epithelial cells and how these cells respond to high oxalate and/or CaOx crystals [4].

Under physiological condition, most of the CaOx crystallizes within urinary tract and is then freely excreted in urine. However, if the crystals are retained within the kidney they have the propensity to grow and develop into stones, which in due course of time lead to injury to the renal epithelial cells and further create a site for the formation of a stone nidus [5]. Under pathological conditions, exposure to high concentrations of oxalate ions and/or CaOx crystals results in toxicity to renal cells. This damage to the renal cells induces alterations in cell surface properties thus, unmasking attachment site for adhesion and/or internalization of crystals by renal epithelial cells [6,7]. The interaction between renal epithelial cells and oxalate and/or CaOx crystals modifies renal cellular functions as well as the extracellular environment, leading to crystal retention and thus, plays a significant role in CaOx stone formation [4].

Treatment and prevention strategies for urolithiasis include combination of surgical procedures, medications and dietary manipulations. Despite the technical advancements for stone removal, problems of recurrence persist [8]. Toxicity, side effects and high cost are the major factors associated with the use of modern synthetic drugs and surgical treatments. To overcome these problems, development of phytotherapeutic agents which can function as an alternate therapy to treat kidney stones is a very attractive option [9]. Various medicinal plants, which are a part of traditional medicine since ancient times, have been utilized as therapeutic remedies as they have been reported to possess antilithiatic activity [10].

The current focus of various pharmaceutical industries is on developing plant- protein based, therapeutic drugs. These phytoproteins could be produced on a large scale and modified by recombinant DNA technology [11] to enhance effectiveness, reduce immunogenicity, allergenic properties as well as toxicity, so as to be safer for use [12]. Most of the plant based antilithiatic proteins identified till date are anionic in nature, and this is due to the presence of acidic amino acids and/or calcium binding domains (EF Hand motifs) [13]. These acidic amino acids bind to the calcium thus preventing its interaction with oxalate and adherence to renal cells [14].

With this background the present study was designed to evaluate the bioactivity of anionic anti-calcifying proteins of dried bark *T*. *arjuna*, on oxalate injured renal epithelial cells, so as to establish a scientific foundation for the anti urolithiatic potential of *T*. *arjuna*. This plant which belongs to the family Combretaceae, is widely used in the traditional system of medicine for various disorders of the cardiovascular system. It is also known to possess antioxidants, anti-inflammatory and mild diuretic activity [15]. Studies have also shown that *T*. *arjuna* inhibits *in vitro* CaOx crystallization and crystal adhesion to renal epithelial cells [16–18]. This study was undertaken to isolate, purify and characterize antilithiatic proteins from the dried bark of *Terminalia arjuna* and assess their influence on different stages of CaOx stone formation. In this study, we present *in vitro* evidence for the presence of four anionic antilithiatic proteins from the bark of *Terminalia arjuna* which could play a crucial role in inhibiting CaOx formation.

## Materials and Methods

### Plant

The dried bark of *Terminalia arjuna* was purchased from Natural Remedies Pvt. Ltd., Bangalore, India. A collection voucher specimen is available at the company.

### CaOx crystallization assay to measure inhibitory activity

Inhibitory activity against CaOx crystal nucleation and aggregation was measured using a time-course measurement of optical density as described previously by Hess et al. [19] with some modifications. Stock solutions of 10.0mM calcium chloride (CaCl_2_) and 1.0 mM sodium oxalate (Na_2_C_2_O_4_), containing 200 mM sodium chloride (NaCl) and 10 mM sodium acetate, were adjusted to pH 5.7. For crystallization experiments, the solutions were warmed up to 37°C followed by addition of 1.5 mL of the CaCl_2_ solution and 1.5 mL of the Na_2_C_2_O_4_ solution in a cuvette to achieve final assay concentrations of 5.0 mM for calcium and 0.5 mM for oxalate, respectively. In the cuvette, the final solutions were stirred continuously and maintained at 37°C and optical density at 620 nm (OD_620_) was recorded after every 60 seconds over 40 minutes. Experiments with protein samples (100 μL) were extended to 60 minutes due to lower rate of nucleation and aggregation. All crystallization experiments were performed thrice in triplicate. Slopes of nucleation (*S*_N_) and aggregation (*S*_A_) phases were calculated using linear regression analysis. Using the slopes, percentage inhibitory activity of protein sample was calculated as (1-(Tsi/Tsc)) x100, where Tsc was the turbidity slope of the control and Tsi the turbidity slope in the presence of the inhibitor.

### CaOx crystal growth assay to measure inhibitory activity

CaOx crystal growth inhibitory activity was measured using the seeded solution-depletion assay described previously by Nakagawa et al. [20] with some modifications. Briefly, aqueous solution 4 mM of CaCl_2_, 4 mM Na_2_C_2_O_4_ and 10 mM Tris-HCl containing 90 mM NaCl (pH 7.2) were prepared and equilibrated at 37°C. Stone slurry (1.5 mg/mL) was prepared in 50 mM sodium acetate buffer (pH 5.7) and 30 μL of stone slurry was added to a solution containing 1 mM CaCl_2_ and 1 mM Na_2_C_2_O_4_. The reaction of CaCl_2_ and Na_2_C_2_O_4_ with crystal seed led to deposition of CaOx crystals on the crystal seed surfaces, reflecting the loss of oxalate due to CaOx crystal growth that is detectable by spectrophotometry at λ214 nm. When protein sample (10 μL) is added into this solution, the consumption of free oxalate to form CaOx crystals will decrease if the sample inhibits CaOx crystal growth. The absorbance was monitored after every 60 seconds for 20 minutes at 214 nm. Rate of depletion of free oxalate was calculated using the baseline value and the value after 60 seconds for 20 minutes, with or without protein sample. The percentage inhibitory activity was calculated as ((C-S)/C) x100, where C is the rate of reduction of free oxalate without a protein sample and S is the rate of reduction of free oxalate with a protein sample.

### Protein extraction and purification from the dried bark of *Terminalia arjuna*

The extraction and purification of proteins from the dried bark of *T*. *arjuna* was conducted as described in [21] with slight modifications. The dried bark of *T*. *arjuna* was ground to fine powder. To obtain whole protein extract, 100 grams of bark powder was extracted with extraction buffer (50 mM Tris-Cl buffer (pH 7.4), containing 0.25 M NaCl, 1 mM Phenylmethylsulfonyl fluoride, 0.01% sodium azide and 5% Polyvinylpyrrolidone). The slurry was stirred continuously for 24 hours at 4°C. After 24 hours, the slurry was centrifuged at 10,000 g for 20 minutes at 4°C. The supernatant was collected and stored at -20°C for further experimentation. This supernatant was referred to as the whole protein extract of *Terminalia arjuna*. Whole protein extract (WPE) of *Terminalia arjuna* was separated into <3 kDa and >3 kDa fractions and dialyzed against 10 mM Tris-Cl buffer at pH 7.4 by centrifugation with the help of Amicon Ultra-4 centrifugal separating tubes (Millipore) of 3 kDa cut off molecular weight. Thus, two fractions <3 kDa and >3 kDa were obtained. Whole protein extract, <3 kDa and >3 kDa fractions of *T*. *arjuna* were assessed for CaOx crystallization and crystal growth inhibitory activity. The >3 kDa fraction exhibited significant inhibitory activity and was loaded on to strong anion exchanger Q Sepharose (GE Healthcare) packed in a column (XK 16/20) equilibrated with 10 mM Tris-Cl buffer (pH 7.4). Bound proteins were eluted by using a linear concentration gradient of NaCl (0–1 M) in 10 mM Tris-Cl buffer (pH 7.4) at a flow rate of 0.5 mL/min. Fractions under each peak were pooled, dialyzed against 10 mM Tris-Cl buffer (pH 7.4) and their inhibitory bioactivity towards CaOx crystal nucleation, aggregation and growth was studied. All the peaks obtained were concentrated and loaded one by one on a Bio gel® P-100 gel molecular sieve column (XK 16/70) equilibrated and eluted with the 10 mM Tris-Cl buffer (pH 7.4) at a flow rate of 0.2 mL/min. The eluted fractions under each peak were pooled to study their activity w.r.t. CaOx crystal nucleation, aggregation and growth as well as on oxalate induced injury to NRK-52E and MDCK cells. The homogeneity of purified proteins was analyzed by Native-PAGE and reverse phase HPLC. Total protein concentration was determined by Bradford Assay at each step of purification using Bovine Serum Albumin (BSA) as a standard [22].

#### Native-PAGE

The purified proteins obtained from molecular sieve chromatography were reconstituted in non-reducing sample buffer and analyzed by native page using 1 mm thick, 12% resolving gel and 5% stacking gel without using SDS or 2-mercaptoethanol (reducing agent) with a Mini-Protean III apparatus (Bio-Rad) at 100 V [23,24]. The gels were stained using silver staining.

#### Homogeneity of purified proteins by High Performance Liquid Chromatography

Homogeneity of purified proteins (A1, B1, B2, C1) was determined by performing reverse phase HPLC using Waters Spherisorb® C18 (5 μm, 4.6 X 250 mm) column with solvent A (0.1% TFA in water) and solvent B (100% acetonitrile containing 0.1% TFA) in a linear gradient of acetonitrile (20–70%) over a period of 50 minutes at a flow rate of 1 mL/min. The sample injection volume was 20 μL and column was washed with solvent A and brought to 20% acetonitrile in 5 minutes. The protein peak was detected at 280 nm using Waters 2996 photodiode array detector [25].

#### Trypsin digestion and peptide mass fingerprinting

The identified bands after analysis were excised and diced into small pieces (1 mm) followed by de-staining using 1:1 (v/v) of potassium ferricyanide and sodium thiosulfate for 10 minutes, and this process was repeated 3–4 times until they become translucent white. They were dehydrated using 100% acetonitrile and Speedvac to complete dryness. The gel pieces were rehydrated with 1.5 mg/mL DTT and incubated for an hour. After incubation, the DTT solution was removed, and further incubated with 10 mg/mL of iodoacetamide for 45 minutes. The supernatant was removed and the gel was dehydrated with 100% acetonitrile for 10 minutes and Speedvac till completely dry. 12 ng/μL trypsin solution was added and incubated overnight at 37°C. The peptides were extracted with 1:1 5% formic acid/H_2_O and final extraction with 1:1 5% formic acid/acetonitrile was performed thrice, followed by Speedvac to complete dryness. The dried peptide mix was suspended in 50% trifluoroacetic acid (TFA) containing 0.1% acetonitrile (ACN) buffer. The peptides obtained were mixed with α-cyano-4-hydroxycinnamic acid (HCCA) matrix in 1:1 ratio and resulting 2 μL was spotted on the MALDI TOF/TOF ULTRAFLEX III instrument and further analysis was done with FLEX ANALYSIS SOFTWARE for obtaining the Peptide Mass Fingerprint.

#### Protein identification and domain prediction

The mass over charge ratio obtained in the peptide mass fingerprinting were submitted for MASCOT search in MASCOT search engine (https://www.matrixscience.com) using SwissProt database for the identification of the proteins. The search parameters used were monoisotopic, oxidized at methionine residues as a variable modification and carbamidomethylated at cysteine residues as a fixed modification. The search was done using a Viridiplantae taxonomy, only one missed tryptic cleavage and peptide mass tolerance of 120 parts per million. The presence of the active domains was found using the online tool, ScanProsite and the amino acid sequence of the hit obtained from MASCOT search was used as an input to search for the presence of active domain.

#### Preparation of protein samples for cell line studies

The >3 kDa fraction and purified proteins obtained after molecular sieve chromatography were dialyzed against distilled water by centrifugation through Amicon Ultra-4 centrifugal separating tubes (Millipore) of 3 kDa cut off molecular weight and then lyophilized. The lyophilized >3 kDa fraction and purified proteins were reconstituted in serum free DMEM and filtered by 0.22 μm syringe filter. Filtered solution of 1.44 mM Tris-Cl buffer (pH 7.4) was used as a solvent system.

### Cell culture

Experimental studies were done using *in vitro* models of Normal rat epithelial derived renal tubular epithelial (NRK-52E) and Madin-Darby Canine Kidney (MDCK) cell lines obtained from NCCS (National Centre for Cell Science), Pune, India. Both the cell lines were maintained in Dulbecco’s Modified Eagle’s Medium (DMEM) supplemented with 10% Fetal Bovine Serum (FBS) and 1% Penicillin (100 units/mL)-Streptomycin (10,000 μg/mL). Cells were cultured in 25 cm^2^ and 75 cm^2^ tissue-culture treated flasks at 37°C under 5% CO_2_ in humidified chambers [26]. The cells were grown to 80% confluency for subsequent subculture in preparation for experiments.

#### Exposure to oxalate

A stock solution of 10 mM sodium oxalate was prepared and diluted to 2 mM in serum-free medium. NRK-52E cells and MDCK cells were exposed to 2 mM sodium oxalate in the absence and presence of different concentrations of >3 kDa fraction and purified proteins (A1, B1, B2, C1) for a period of 48 hours [27,28]. Cystone drug at a concentration of 10 μg/mL was used as a positive control.

#### MTT assay

1x10^4^ cells were seeded into each well of a 96-well microplate and incubated at 37°C and 5% CO_2_ in humidified chambers. At 80% confluency, cells were treated with 2 mM sodium oxalate in the absence and presence of different concentrations (4 μg/mL, 6 μg/mL, 8 μg/mL, 10 μg/mL) of >3 kDa fraction and purified proteins (A1, B1, B2, C1) for 48 hours at 37°C. At the end of the treatment, 25 μL of MTT reagent (final concentration of 0.5 mg/mL) was added to each well and incubated for 4 hours at 37°C. After incubation, medium was replaced with 200 μL DMSO (100%) and kept still at room temperature for 15–20 minutes. After gentle mixing, absorbance values were determined at a 570 nm test wavelength and a 630 nm reference wavelength to evaluate the cell viability using a microplate reader (Model 680, Bio-Rad) [29].

#### CaOx crystal adhesion

Cells were seeded on sterile glass coverslips placed in a 6-well plate at a density of 2x10^5^ cells/coverslip and cultured at 37°C and 5% CO_2_ in humidified chambers to achieve 80% confluency. After incubation, cells were treated with 2 mM sodium oxalate in the absence and presence of >3 kDa fraction and purified proteins (A1, B1, B2, C1) at a concentration of 10 μg/mL for 48 hours at 37°C. After the treatment, medium was removed and cells were washed twice with 1X PBS followed by fixation of cells with 4% paraformaldehyde for 30 minutes. After incubation, cells were again washed twice with 1X PBS and then observed under phase contrast and polarization upright microscope (BX53, Olympus Corporation, Japan) at a magnification of 20X to study cell-crystal interactions [30].

#### Hoechst 33258 staining

Cells were seeded on sterile glass coverslips placed in a 6-well plate at a density of 2x10^5^ cells/coverslip and cultured at 37°C and 5% CO_2_ in humidified chambers to achieve 80% confluence. After incubation, cells were treated with 2 mM sodium oxalate in the absence and presence of >3 kDa fraction and purified proteins (A1, B1, B2, C1) at a concentration of 10 μg/mL for 48 hours at 37°C. At the end of treatment, medium was removed and cells were washed twice with 1X PBS followed by fixation of cells with 4% paraformaldehyde for 30 minutes. After incubation, cells were washed twice with 1X PBS, stained with 5 μg/mL of Hoechst 33258 dye for 10 minutes at room temperature in the dark and again washed twice with 1X PBS. Stained nuclei were observed under fluorescence upright microscope (BX53, Olympus Corporation, Japan) at a magnification of 20X [31].

#### Annexin V/ propidium iodide staining

6x10^5^ cells were seeded into 60 mm dishes and incubated at 37°C and 5% CO_2_ in humidified chambers. At 80% confluency, cells were treated with 2 mM sodium oxalate in the absence and presence of >3 kDa fraction and purified proteins (A1, B1, B2, C1) at a concentration of 10 μg/mL for 48 hours at 37°C. After treatment, the subsequent procedure followed was in accordance to the instructions of BD Pharminogen^TM^ FITC ANNEXIN V Apoptosis Detection Kit 1 (catalogue no. 556547), where, cell suspension and cells from monolayer were pooled together. The cells were washed with cold 1X PBS twice. The pellet was resuspended in 100 μL of 1X binding buffer followed by addition of 2 μL of FITC Annexin V and 2 μL of Propidium iodide (PI). The cells were gently vortexed and incubated for 15 minutes at room temperature in the dark. 400 μL of 1X binding buffer was added to each group and the cells analyzed by flow cytometry (BD Accuri C6, BD Biosciences).

#### Detection of active caspase-3

6x10^5^ cells were seeded into 60 mm dishes and incubated at 37°C and 5% CO_2_ in humidified chambers. At 80% confluency, cells were treated with 2 mM sodium oxalate in the absence and presence of >3 kDa fraction and purified proteins (A1, B1, B2, C1) at a concentration of 10 μg/mL for 48 hours at 37°C. At the end of the treatment, the subsequent procedure followed was in accordance to the instructions of BD Pharminogen^TM^ FITC Active Caspase-3 Apoptosis Kit (catalogue no. 550480), where, cell suspension and cells from monolayer were pooled together. Cells were washed with cold PBS twice and resuspended in BD Cytofix/Cytoperm solution. After incubation on ice for 20 minutes, BD Cytofix/Cytoperm solution was discarded. The cells were washed twice with 1X BD Perm/Wash buffer at room temperature. The cells were then resuspended in the 1X BD Perm/Wash buffer plus 10 μL of antibody and incubated for 30 minutes at room temperature. After incubation, the cells were washed with 0.5 mL of 1X BD Perm/Wash buffer and then resuspended in 0.5 mL of 1X BD Perm/Wash buffer for analysis by flow cytometry (BD Accuri C6, BD Biosciences).

### Statistical analysis

Statistical procedures were performed with GraphPad Prism software version 6.01. The statistically different groups were identified by one-way analysis of variance (ANOVA), followed by Dunnet’s multiple comparisons test. Results were expressed as the mean ± SD. A p-value of <0.05 was considered significant. All the experiments were performed three times, each time in triplicate.

## Results

### CaOx crystal nucleation, aggregation and growth inhibitory activity of whole protein extract, <3 kDa and >3 kDa fractions

**Fig 1:**
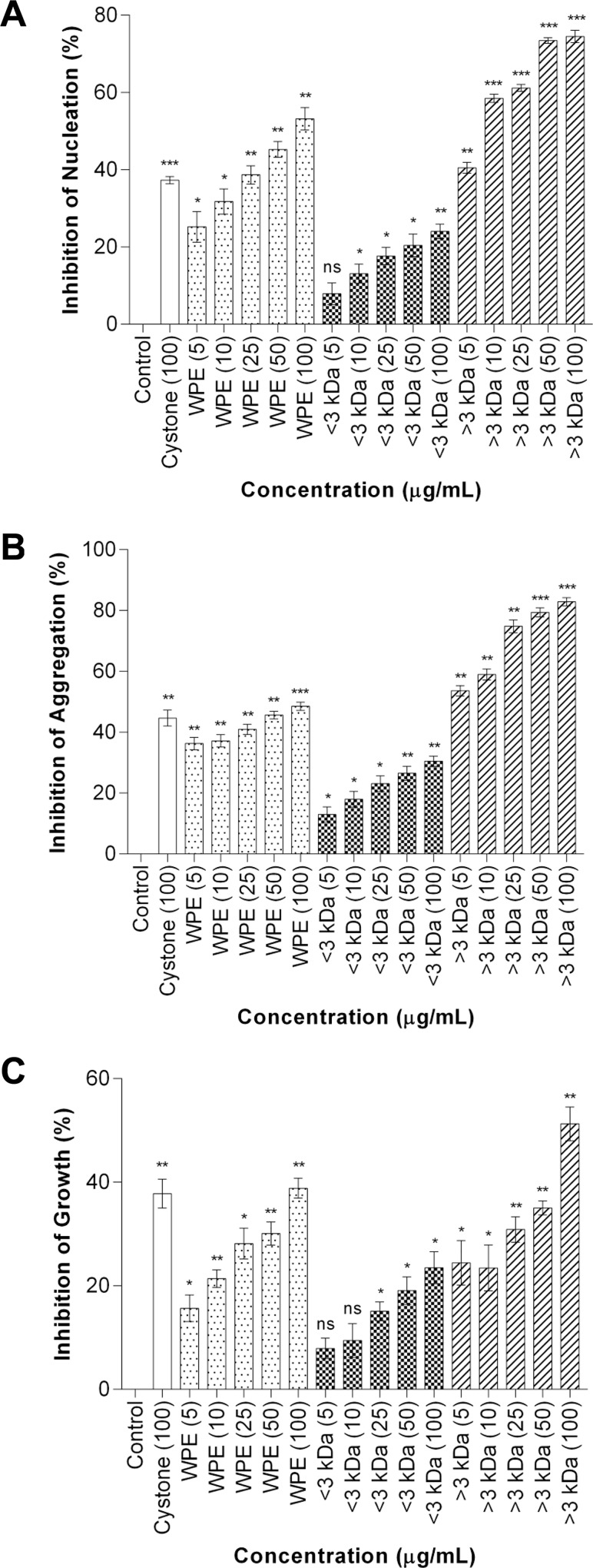
CaOx crystallization inhibitory activity of WPE, < 3kDa and > 3kDa fractions. Effect of WPE, <3 kD and >3 kDa fraction on calcium oxalate crystallization. (A)Nucleation. (B)Aggregation fraction. (C)Growth. Data are mean ± S.D of three independent observations. The statistically different groups were identified by one-way analysis of variance (ANOVA), followed by Dunnet’s multiple comparisons test. * p < 0.05 vs control, ** p < 0.01 vs control, *** p < 0.001 vs control and ‘ns’ represents not significant.

Inhibitory activity of whole protein extract, <3 kDa fraction and >3 kDa fraction was studied individually against CaOx crystal nucleation, aggregation and growth kinetics and inhibitory activity wherein a dose dependent effect was seen. Although the whole protein extract and <3 kDa fraction showed inhibitory activity towards CaOx crystal nucleation (Fig 1A), aggregation (Fig 1B) and growth assay (Fig 1C) system, however, the >3 kDa fraction exhibited maximum inhibitory activity (Fig 1A, 1B and 1C) and was taken further for bioactivity guided purification.

### Purification of antilithiatic proteins

**Fig 2:**
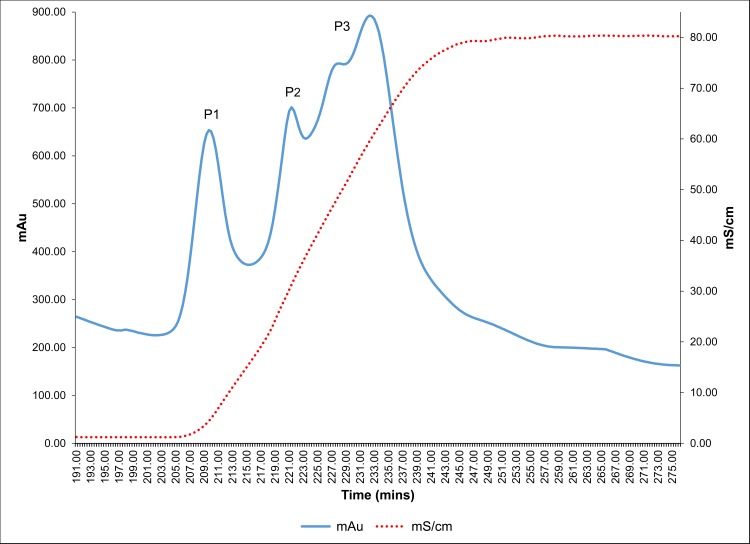
Anion exchange chromatography profile of >3 kDa fraction. Elution profile of >3 kDa fraction of *T*. *arjuna* loaded on Q Sepharose anion exchanger, with a linear gradient of NaCl (0–1 M).

**Fig 3:**
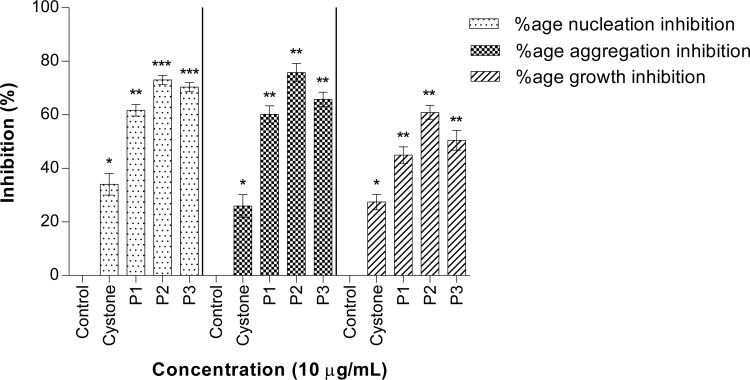
CaOx crystallization inhibitory activity of peaks P1, P2 and P3. Percentage inhibitory activity of peaks of anion exchange chromatography on nucleation, aggregation and growth of CaOx crystals. Data are mean ± S.D of three independent observations. All treatment groups were simultaneously compared via one-way ANOVA using Dunnett’s multiple comparisons test. * p < 0.05 vs control, ** p < 0.01 vs control, *** p < 0.001 vs control.

In order to purify the antilithiatic proteins from the > 3 kDa fraction of *T*. *arjuna* a multi- step protocol involving anion exchange and gel filtration chromatography, followed by Native-PAGE was undertaken (S1 Fig). On subjecting the > 3 kDa fraction to an anion exchanger Q Sepharose column, successive fractions under each peak with increasing gradient of NaCl were collected, pooled and named P1 to P3 (Fig 2). These eluted peaks were dialyzed against 10 mM Tris-Cl buffer (pH 7.4) and it was seen that peaks P1, P2 and P3 exhibited significant inhibitory activity against CaOx crystal nucleation, aggregation and growth kinetics (Fig 3). The order of inhibitory activity was peak P2 > peak P3 >peak P1 at a concentration of 10 μg/mL.

**Fig 4:**
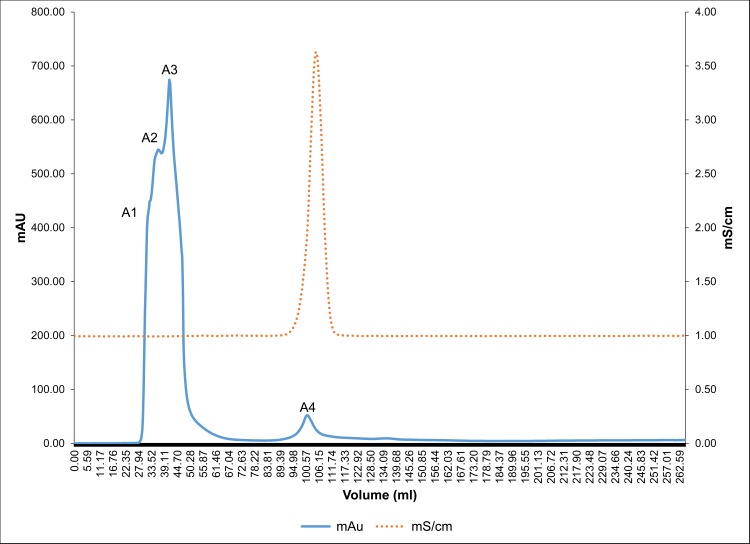
Gel filtration chromatography profile of peak P1. Chromatogram of peak P1 subjected to Bio gel^®^ P-100 gel packed column and proteins eluted in an isocratic 10 mM Tris-Cl buffer (pH 7.4).

**Fig 5:**
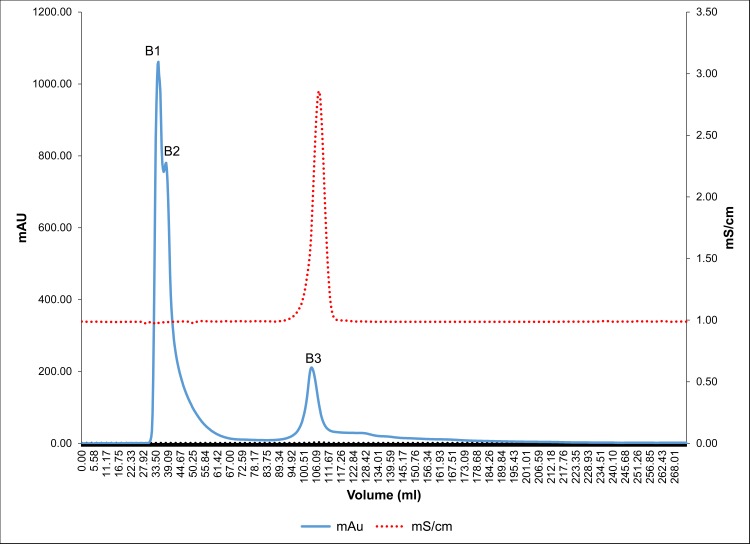
Gel filtration chromatography profile of peak P2. Chromatogram of peak P2 subjected to Bio gel^®^ P-100 gel packed column and proteins eluted in an isocratic 10 mM Tris-Cl buffer (pH 7.4).

**Fig 6:**
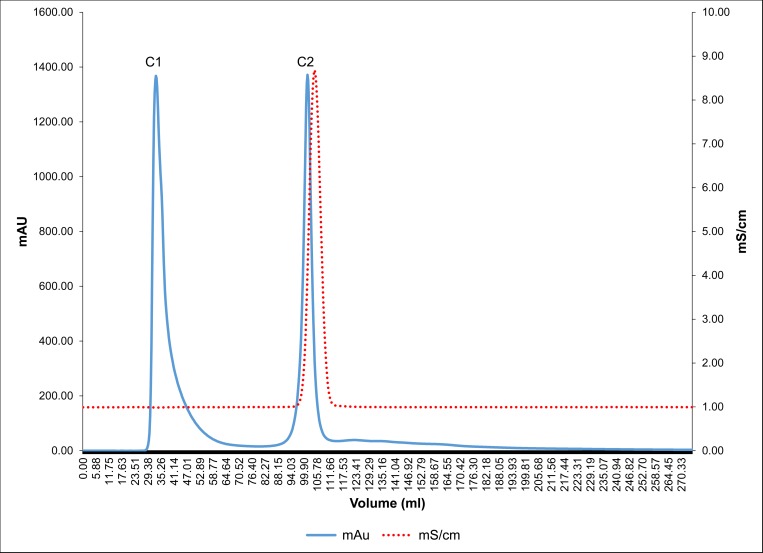
Gel filtration chromatography profile of peak P3. Chromatogram of peak P3 subjected to Bio gel^®^ P-100 gel packed column and proteins eluted in an isocratic 10 mM Tris-Cl buffer (pH 7.4).

**Fig 7:**
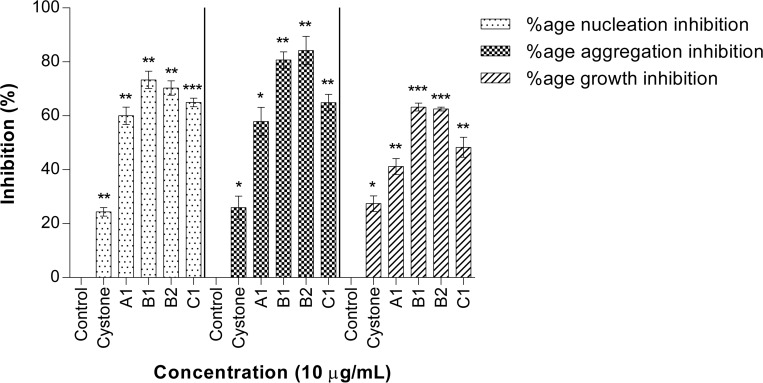
CaOx crystallization inhibitory activity of eluted purified proteins. Percentage inhibitory activity of most active peaks obtained from gel filtration chromatography on nucleation, aggregation and growth of CaOx crystals. Data are mean ± S.D of three independent observations. All treatment groups were simultaneously compared via one-way ANOVA using Dunnett’s multiple comparisons test. * p < 0.05 vs control, ** p < 0.01 vs control, *** p < 0.001 vs control.

Anionic protein separation was followed by molecular separation of proteins by gel filtration chromatography. Peaks P1, P2 and P3 were individually loaded (Figs 4, 5 and 6) on a Bio gel^®^ P-100 gel packed column (XK 16/70) and proteins were eluted isocratically with 10 mM Tris-Cl buffer (pH 7.4). On subjecting peak 1, peak 2 and peak 3 to gel filtration chromatography the subsequent fractions obtained were pooled and named as A1 to A4, B1 to B3 and C1 to C2, respectively. These peaks were then assessed for their inhibitory activity and it was seen that peaks A1, B1, B2, C1 exhibited the maximum inhibitory potential against CaOx crystal nucleation, aggregation and growth kinetics (Fig 7). We observed appreciable inhibition with 10 μg/ml in the > 3kDa fraction which basically contained a number of proteins, and following the purification procedure, the specific activity of these proteins towards inhibition of nucleation, aggregation and growth was more pronounced.

**Fig 8:**
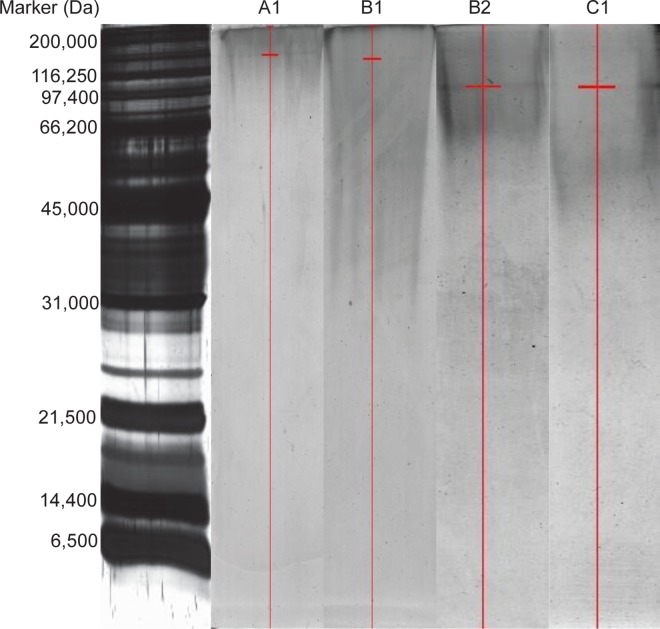
Native-PAGE analysis. Native-PAGE of most active peaks with maximum inhibitory activity obtained from gel filtration chromatography. The short red horizontal line indicates the position of the purified protein band. Lane 1: Broad range marker (Biorad), Lane 2: A1, Lane 3: B1, Lane 4: B2 and Lane 5: C1.

**Fig 9:**
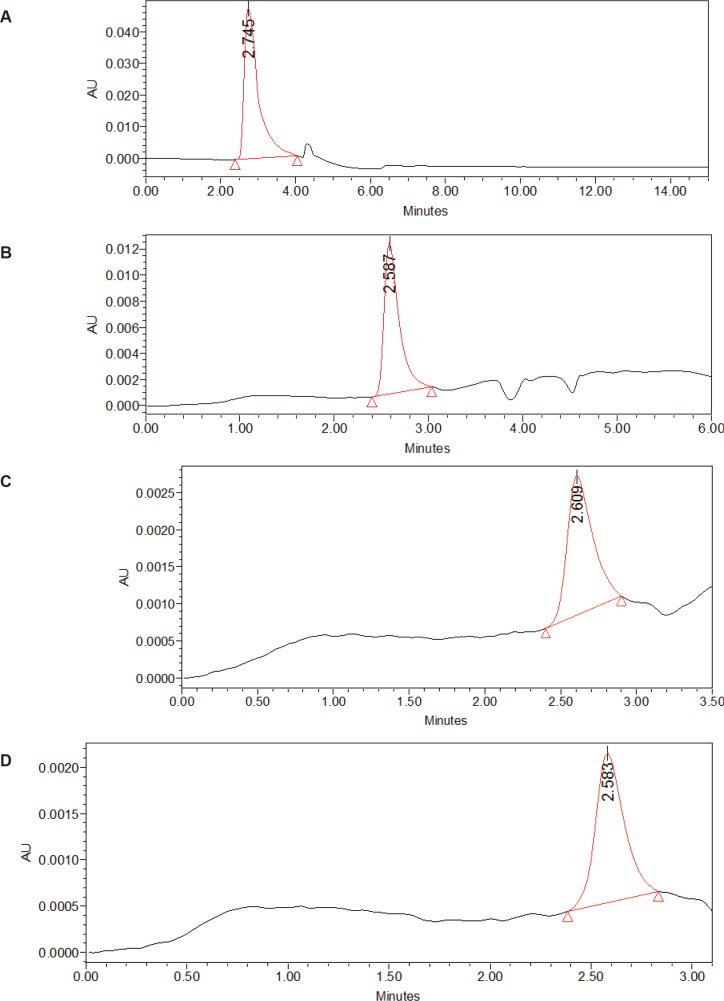
Homogeneity of purified proteins by HPLC analysis. Reverse phase HPLC analysis of purified protein (A) A1. (B) B1. (C) B2. (D) C1.

The purified peaks A1, B1, B2, C1 exhibiting marked inhibitory activity were subjected to Native-PAGE (Fig 8) wherein single bands of MW ~190 kDa, ~130 kDa, ~90 kDa and ~90 kDa, respectively, were seen. Homogeneity of purified proteins was further verified using RP-HPLC, wherein single peaks were observed (Fig 9).

### MALDI-TOF Mass spectrometric analysis and identification of purified proteins

The purified proteins obtained were subjected to MALDI-TOF MS and MASCOT search engine analysis. The mass over charge ratio data obtained from the MALDI-TOF of the peaks A1, B1, B2, C1 matched significantly with Nuclear pore anchor, DEAD Box ATP-dependent RNA helicase 45, Lon protease homolog 1 and Heat shock protein 90–3, respectively (Table 1). The amino acid sequence of these respective proteins obtained from MASCOT search was used as an input to search for the presence of active domain using ScanProsite (Table 1).

**Table 1:** Summary of proteins identified and characterized using the Mascot search engine and ScanProsite.

Purified protein peaks	Significant protein match from MASCOT search engine	Matching Score	Sequence Coverage	Molecular Weight (Da)	pI	Active Domains/Motifs from ScanProsite
A1	Nuclear pore anchor	50	15	237,637	5.01	• Lysine rich region • Glutamic acid rich region
B1	DEAD Box ATP-dependent RNA helicase 45	33	14	105,223	5.14	• Glutamic acid rich region • Aspartic acid rich region • Alanine rich region
B2	Lon protease homolog 1	49	22	104,379	5.42	• Cell attachment sequence i.e. RGD (Arg-Gly-Asp)
C1	Heat shock protein 90–3	27	11	80,287	4.95	• Glutamic acid rich region

### Protective effect of purified proteins against oxalate-induced renal epithelial cell injury (MTT assay)

**Fig 10:**
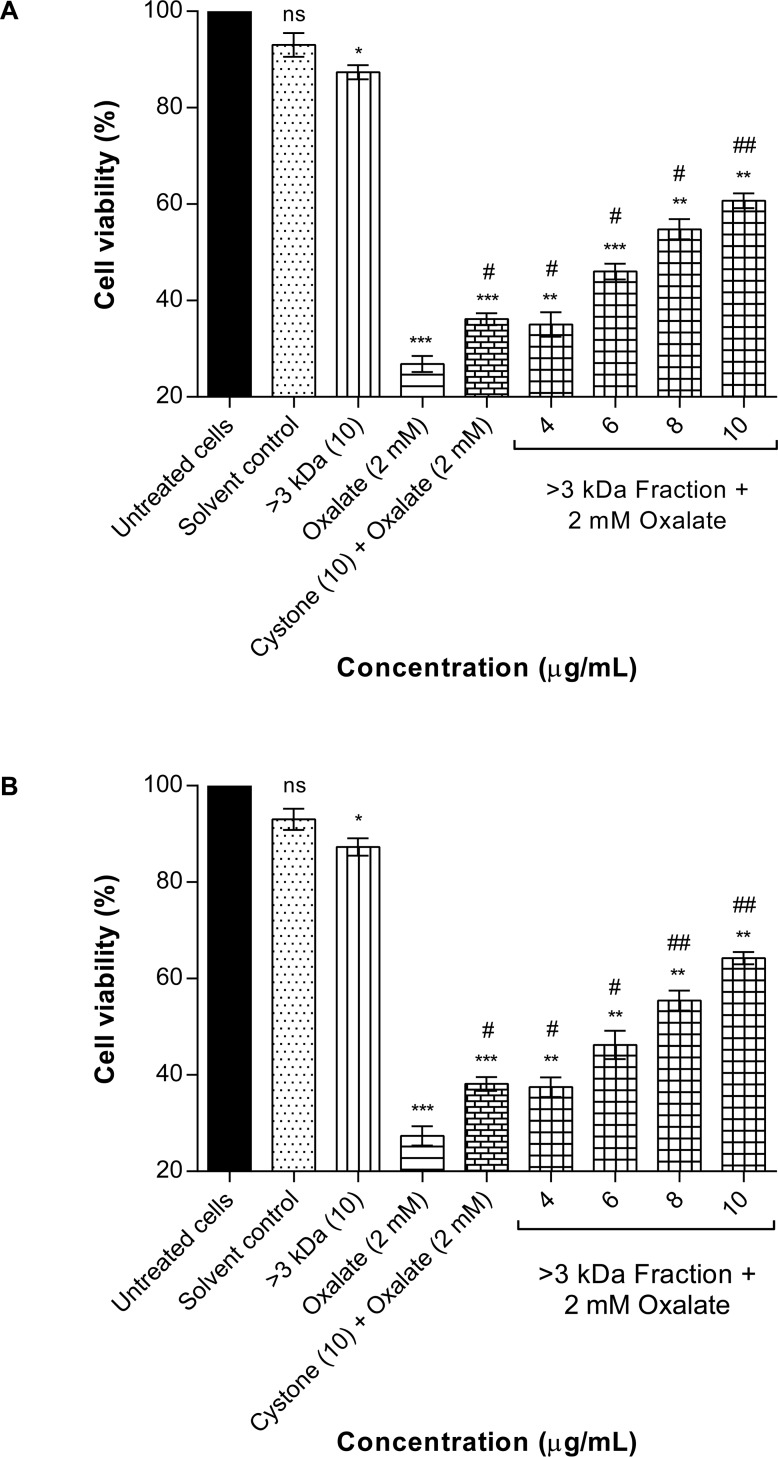
Protective effect of >3 kDa fraction on oxalate injured renal cells (MTT assay). (A) Assessment of viability of oxalate injured NRK-52E cells in the presence of >3 kDa fraction of *T*. *arjuna* by MTT assay. Data are mean ± S.D of three independent observations. ns: not significant. All treatment groups were simultaneously compared via one-way ANOVA using Dunnett’s multiple comparisons test. ‘*’ represents P values versus untreated cells (control) and ‘#’ represents P values versus oxalate treated cells, where * p< 0.05 vs control, ** p < 0.005 vs control, *** p < 0.001 vs control, # p < 0.05 vs oxalate, ## p < 0.01 vs oxalate. (B) Assessment of viability of oxalate injured MDCK cells in the presence of >3 kDa fraction of *T*. *arjuna* by MTT assay. Data are mean ± S.D of three independent observations. ns: not significant. All treatment groups were simultaneously compared via one-way ANOVA using Dunnett’s multiple comparisons test. ‘*’ represents P values versus untreated cells (control) and ‘#’ represents P values versus oxalate injured cells, where * p< 0.05 vs control, ** p < 0.005 vs control, *** p < 0.001 vs control, # p < 0.05 vs oxalate, ## p < 0.005 vs oxalate.

**Fig 11:**
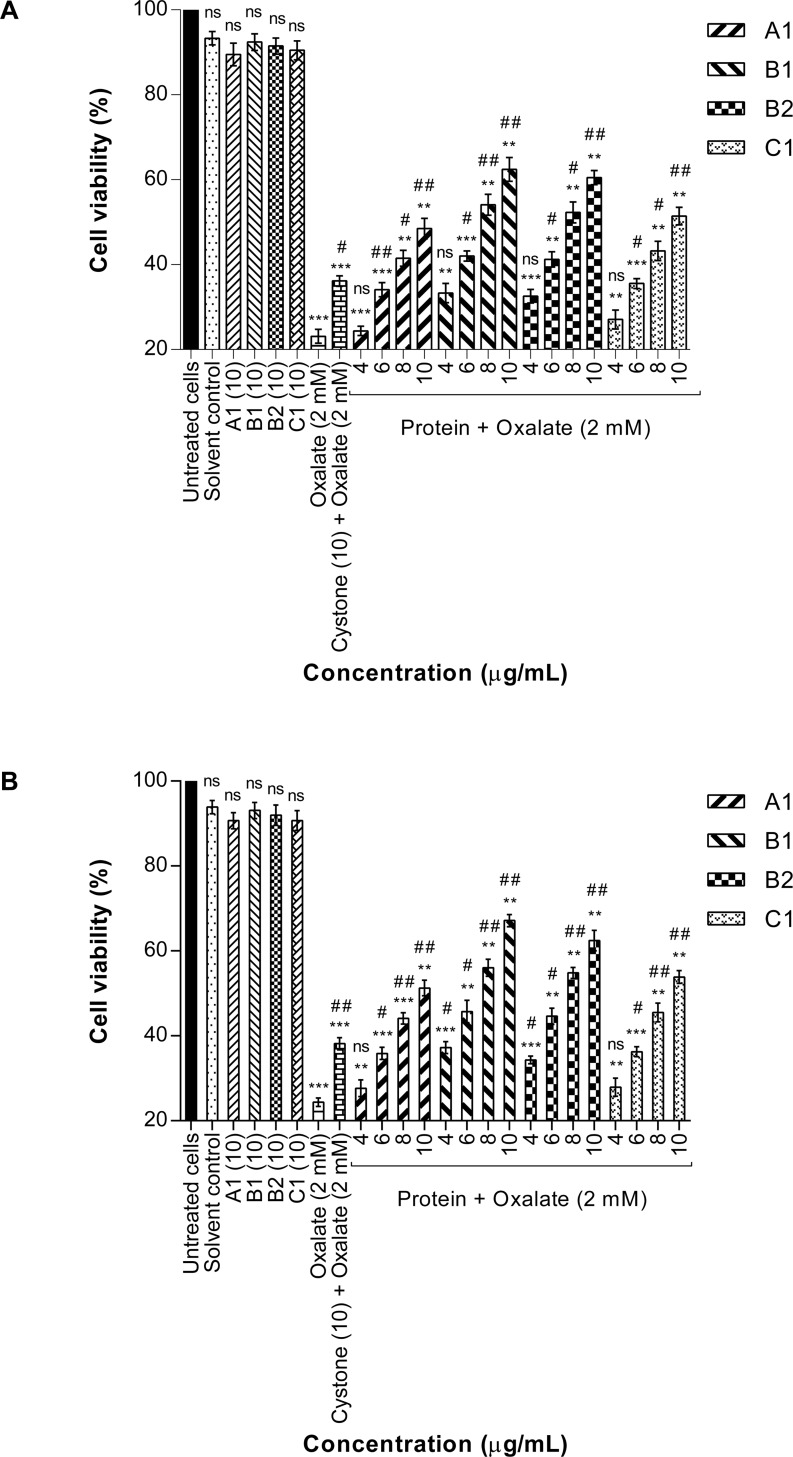
Protective effect of proteins on oxalate injured renal cells (MTT assay). (A) Effect of purified proteins of *T*. *arjuna* on oxalate injured NRK-52E cell viability assessed by MTT assay. Data are mean ± S.D of three independent observations. ns: not significant. All treatment groups were simultaneously compared via one-way ANOVA using Dunnett’s multiple comparisons test. ** p< 0.01 vs control, *** p < 0.001 vs control and # p < 0.05 vs oxalate, ## p < 0.01vs oxalate. (B) The effect of purified proteins of *T*. *arjuna* on oxalate injured MDCK cell viability assessed by MTT assay. Data are mean ± S.D of three independent observations. ns: not significant. All treatment groups were simultaneously compared via one-way ANOVA using Dunnett’s multiple comparisons test. ‘*’ represents P values versus untreated cells (control) and ‘#’ represents P values versus oxalate injured cells l, ** p< 0.01vs control, *** p < 0.001vs control; and # p < 0.05 vs oxalate, ## p < 0.01vs oxalate.

The protective effect of >3 kDa fraction (Fig 10) and purified proteins (A1, B1, B2, C1) (Fig 11) of *Terminalia arjuna* against oxalate induced injury to NRK-52E cells and MDCK cells was evaluated after 48 hours of treatment, by measuring the reduction of yellow tetrazolium to purple formazan crystals. Cells treated with serum free medium were taken as the untreated control group. On exposing the renal epithelial cells to either the solvent system (1.44 mM Tris-Cl buffer pH 7.4) or >3 kDa fraction (10 μg/mL) or the purified proteins (10 μg/mL), no significant effect on the cell viability was observed. The dose of 10 μg/ml was chosen on the basis of studies carried out earlier in our lab using

> 3kDa and protein fractions isolated from other plants wherein, we had seen protection against oxalate induced cell injury. Based on our previous findings we tested various concentrations of > 3kDa and protein fractions of *Terminalia arjuna*, however, at concentrations beyond 10ug/ml protection against oxalate injury was lost, resulting in a decrease in cell viability (data not shown). This cell killing could be attributed to an increase in the concentration of salts in the protein fractions, as the extraction procedure was carried out in Tris-Cl buffer.

Marked cell death was seen on exposure to 2 mM oxalate wherein, there was a sharp decline in viability from 100% in untreated cells to 23.13 ± 1.64% (p<0.001) in NRK-52E cells and 24.36 ± 0.97% (p<0.001) in MDCK cells, respectively. When the oxalate injured cells were co-treated for 48 hours with either the >3 kDa fraction or the purified proteins, the viability significantly increased in a concentration dependent manner. Cystone at a concentration of 10 μg/mL was used as a positive control and which was also shown to protect the cells from oxalate induced injury.

### Loss of CaOx crystal adherence to renal tubular epithelial cells by purified proteins

**Fig 12:**
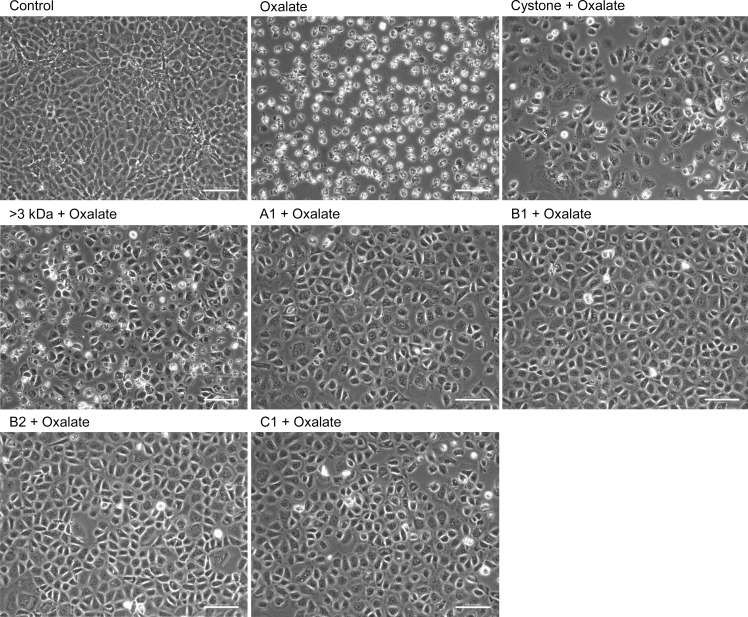
Decreased CaOx crystal adhesion to NRK-52E cells by proteins. Effect of >3 kDa fraction and purified proteins of *T*. *arjuna* on CaOx crystal adherence to oxalate injured NRK-52E cells, visualized under polarization and phase contrast at magnification 20X and scale bar 100 microns.

**Fig 13:**
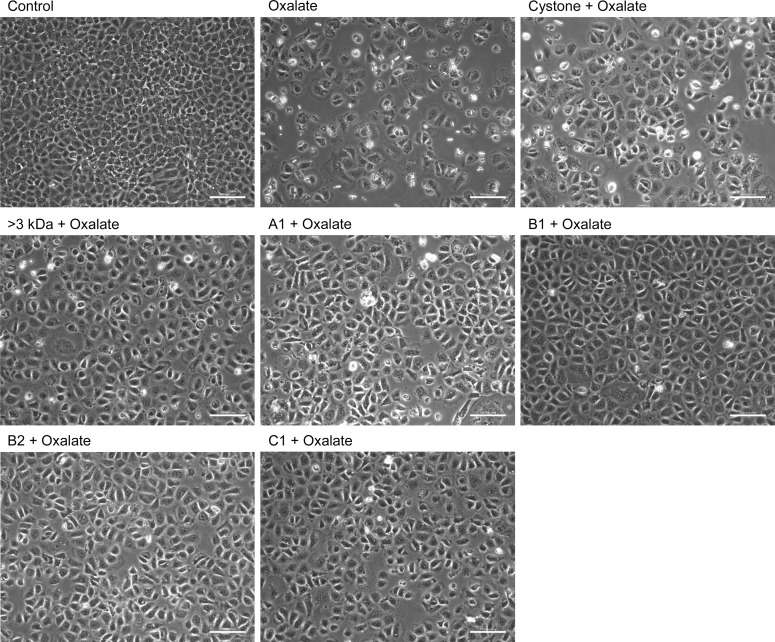
Decreased CaOx crystal adhesion to MDCK cells by proteins. Effect of >3 kDa fraction and purified proteins of *T*. *arjuna* on CaOx crystal adherence to oxalate injured MDCK cells, visualized under polarization and phase contrast at magnification 20X and scale bar 100 microns.

To see the manner in which oxalate injures the renal epithelial cells, CaOx crystal adhesion to renal cell surface of was studies in both the NRK-52E cells (Fig 12) and MDCK cells (Fig 13) 48 hours after treatment. Cells incubated with serum free defined medium were considered as untreated cells i.e. control group. In both the cell lines, cells treated with the solvent system, >3 kDa fraction and purified proteins (A1, B1, B2, C1) showed healthy cellular morphology, indicating that there was no adverse effect to the cells (data not shown). Upon exposure of NRK-52E cells and MDCK cells to oxalate, a marked change in the morphology of the cells was apparent with a decrease in size, increase in the granularity and lesser number of cells attached to the dish with a number of dead cells floating in the medium. In addition, COM crystals were observed microscopically, when the renal cells were treated with 2mM sodium oxalate, and these crystals remained adhered tightly to the cells even after several washes using PBS. The adhesion of the crystals led to cellular damage and eventually cell death, which was reflected by a concomitant decrease in the number of viable cells, as compared to untreated cells. The treatment of oxalate injured NRK-52E and MDCK cells with 10 μg/mL of >3 kDa fraction or purified proteins (A1, B1, B2, C1) disrupted the interaction between cells and crystals, showing loss of crystal adherence to cells, which could be visualized from the polarization micrographs, in which very few crystals could be seen as compared to the oxalate injured cells. On observing the morphology of the cells, they appeared to be similar in morphology to the control cells and further was an increase in the number of viable cells attached in the culture dish. Treatment with Cystone also exhibited similar cytoprotective effects.

### Hoechst staining of oxalate injured renal tubular epithelial cells to detect apoptosis

**Fig 14:**
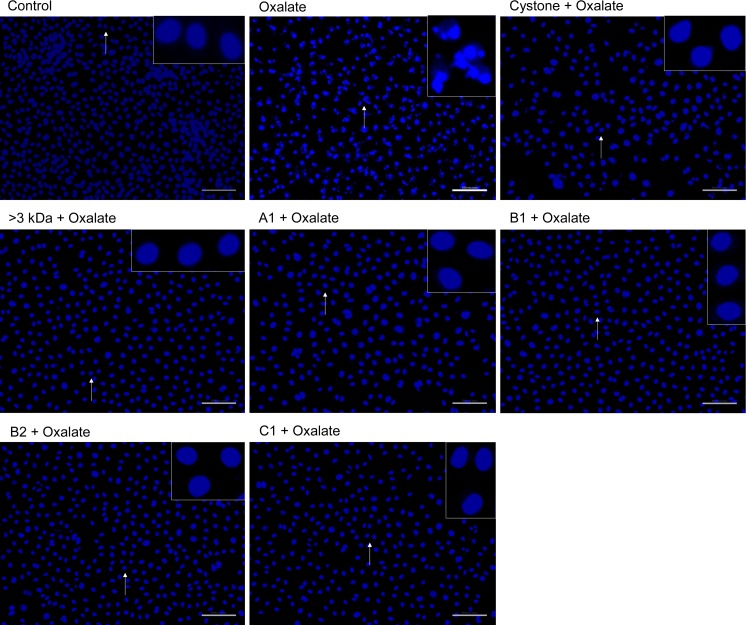
Detection of oxalate induced apoptosis in NRK-52E cells by Hoechst staining. Effect of >3 kDa fraction and purified proteins of *T*. *arjuna* on oxalate induced apoptosis in NRK-52E cells, visualized under fluorescence microscopy at magnification 20X; scale bar 100 microns.

**Fig 15:**
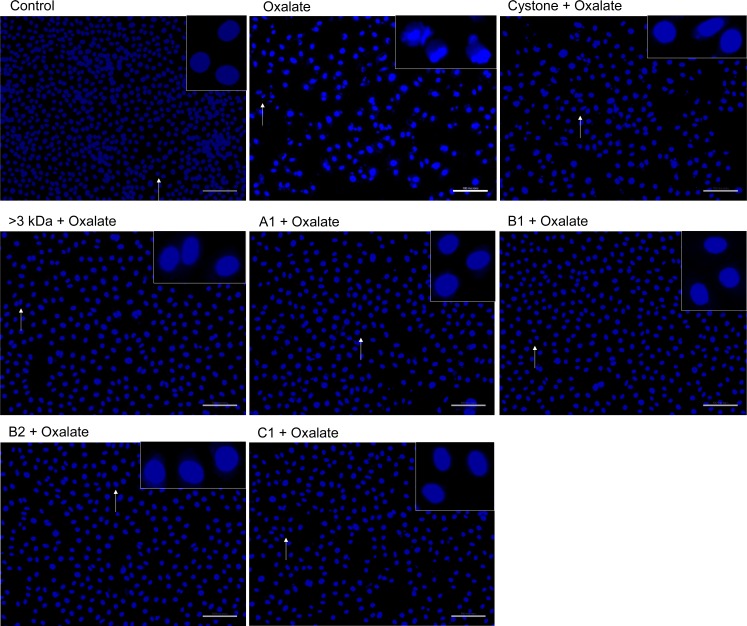
Detection of oxalate induced apoptosis in MDCK cells by Hoechst staining. Effect of >3 kDa fraction and purified proteins of *T*. *arjuna* on oxalate induced apoptosis in MDCK cells, visualized under fluorescence microscopy at magnification 20X; scale bar 100 microns.

To ascertain the nuclear changes, NRK-52E cells (Fig 14) and MDCK cells (Fig 15) were stained with Hoechst 33258 dye. The untreated NRK-52E and MDCK nuclei reflected a healthy morphology with intact chromatin. Treatment of cells with the solvent system, >3 kDa fraction and purified proteins (A1, B1, B2, C1) in the absence of any oxalate damage caused no harmful effects to cells w.r.t. control and nuclei showed intact chromatin (data not shown). However, when both the cell lines were exposed to oxalate, marked changes were observed w.r.t. untreated control group. The nuclear changes reflected signs fragmentation (inset) and were brightly stained, indicative of early apoptotic changes. The effect of 10 μg/mL of >3 kDa fraction and purified proteins (A1, B1, B2, C1) on oxalate treated NRK-52E cells and MDCK cells was assessed and it was apparent that treatment protected the cells as nuclear morphology was similar to the untreated cells. Similar changes were seen upon treatment with Cystone, which also lead to more viable cells as compared to oxalate injured cells.

### Reduction of oxalate-induced apoptosis in renal tubular epithelial cell by purified proteins

**Fig 16:**
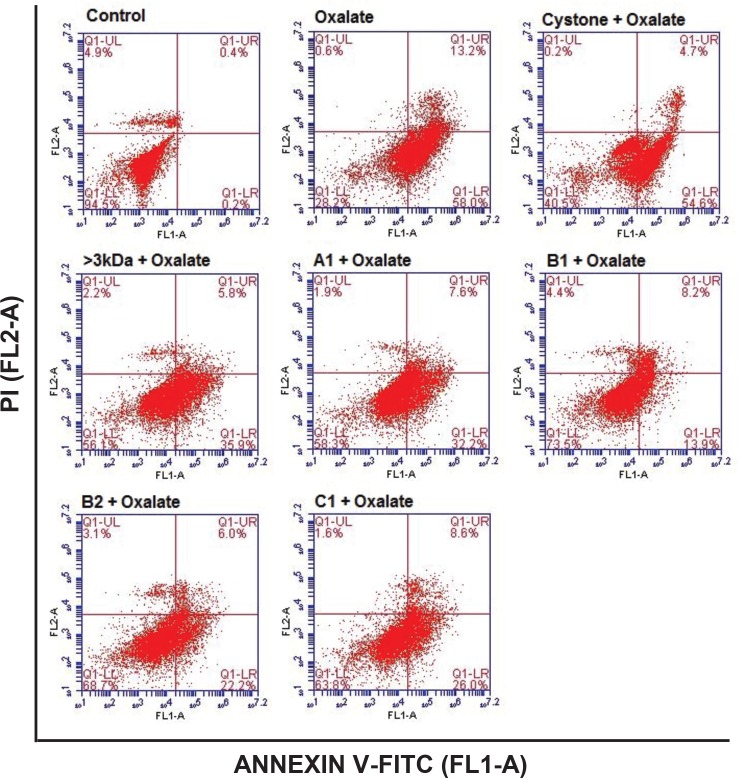
Attenuation of apoptotic cell death in NRK-52E cells by *T*. *arjuna* proteins. Flow cytometry analysis showing the effect of >3 kDa and purified proteins of *T*. *arjuna* on oxalate induced apoptosis in NRK-52E cells, visualized by AnnexinV/PI staining.

**Fig 17:**
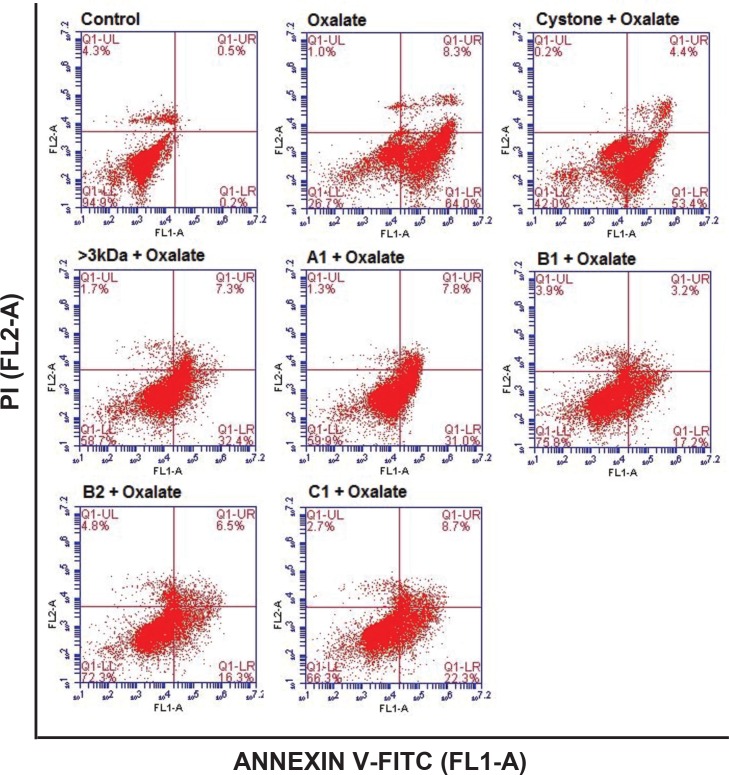
Attenuation of apoptotic cell death in MDCK cells by *T*. *arjuna* proteins. Flow cytometry analysis showing the effect of >3 kDa and purified proteins of *T*. *arjuna* on oxalate induced apoptosis in MDCK cells, visualized by Annexin V/PI staining.

Oxalate-induced apoptosis was assessed after 48 hours in NRK-52E cells (Fig 16) and MDCK cells (Fig 17) using Annexin V/PI staining by flow cytometry. There was no significant apoptosis in the untreated NRK-52E cells and MDCK cells as the maximum number of cells were present in the lower left hand quadrant indicative of a live cell population. Treatment of cells with either the solvent system, or >3 kDa fraction or purified proteins (A1, B1, B2, C1) showed a similar trend (data not shown). Following treatment of the NRK-52E and MDCK cells with oxalate, the percent cell death (early apoptosis, lower right hand quadrant) increased from 0.2% in control to 58% in NRK-52E cells and 64% in MDCK cells. The number of late apoptotic cells (top right hand quadrant) also increased in comparison to the respective controls to 13.2% in NRK-52E and 8.3% in MDCK cells. Treatment with 10 μg/mL of >3 kDa fraction or purified proteins (A1, B1, B2, C1) on oxalate injured NRK-52E and MDCK cells significantly reduced the number of apoptotic cells in comparison to the oxalate treatment alone to 35.9% and 32.2%, 13.9%, 22.2% and 26% in NRK-52E cells and to 32.4% and 31%, 17.2%, 16.3% and 22.3% in MDCK cells, respectively. These results, alluded to the fact that >3 kDa fraction and purified proteins protected the cells from oxalate induced apoptosis. The addition of Cystone to oxalate treated renal cells also improved cell viability in comparison to oxalate injured cells from 28.2% to 40.5% in NRK-52E cells and from 26.7% to 42% in MDCK cells, respectively.

**Fig 18:**
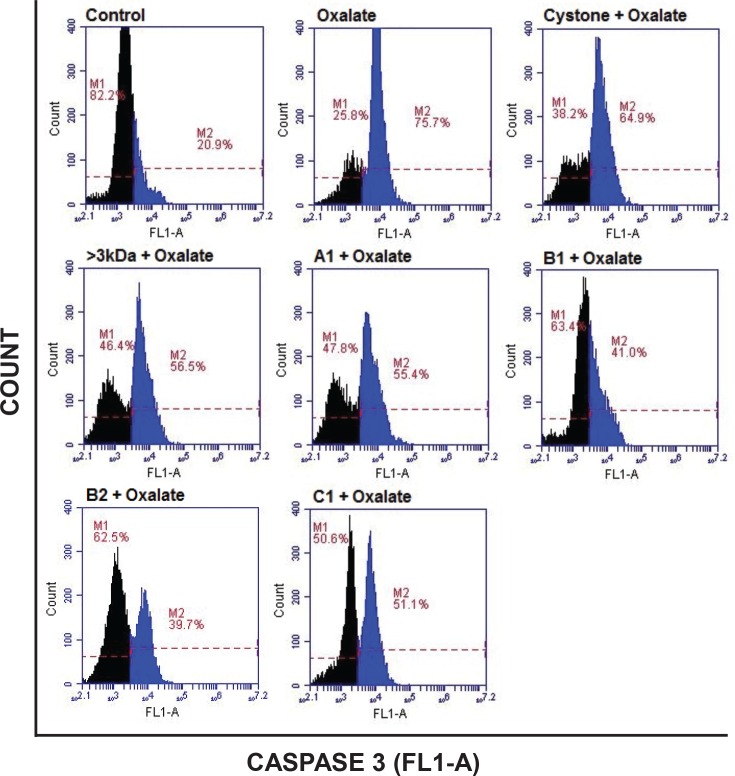
Diminution of Active Caspase-3 in oxalate injured NRK-52E cells. Flow cytometry analysis showing the effect of >3 kDa and purified proteins of *T*. *arjuna* on oxalate induced apoptosis in NRK-52E cells, visualized by Anti-Active Caspase-3 antibody staining. M1 depicts the percentage of viable cells within the margins of marker M1; M2 depicts the percentage of cells undergoing apoptosis within the margins of marker M2(shown in blue).

**Fig 19:**
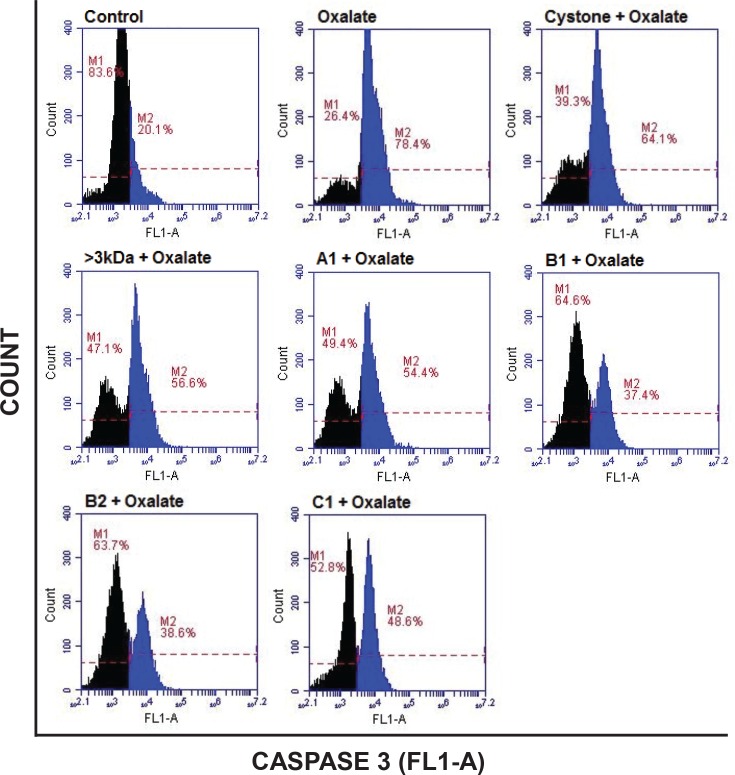
Diminution of Active Caspase-3 in oxalate injured MDCK cells. Flow cytometry analysis showing the effect of >3 kDa and purified proteins of *T*. *arjuna* on oxalate induced apoptosis in MDCK cells, visualized by Anti-Active Caspase-3 antibody staining. M1 depicts the percentage of viable cells within the margins of marker M1; M2 depicts the percentage of cells undergoing apoptosis within the margins of marker M2(shown in blue).

Oxalate-induced apoptosis in NRK-52E cells (Fig 18) and MDCK cells (Fig 19) was further evaluated by anti-active Caspase-3 antibody, which preferentially stains the cells undergoing apoptosis. When both the cell lines were treated with oxalate for a period of 48 hours, the cells undergoing apoptosis increased from 20.9% in control to 75.7% in NRK-52E cells and from 20.1% in control to 78.4% in MDCK cells, respectively. The treatment of 10 μg/mL of >3 kDa fraction or purified proteins A1, B1, B2 and C1 on oxalate injured cells significantly reduced the number of cells expressing active caspase-3 enzyme to 56.5% and 55.4%, 41%, 39.7% and 51.1% in NRK-52E cells and to 56.6% and 54.4%, 37.4%, 38.6% and 48.6% in MDCK cells, respectively, indicating that >3 kDa fraction and purified proteins diminished oxalate induced apoptosis to the renal cells. Although cystone treatment on oxalate injured renal cells did reduce the level of apoptosis to 64.9% in NRK-52E cells and to 64.1% in MDCK cells, respectively, however, the effect was less as compared to the injured cells treated with the purified proteins.

## Discussion

Studies using cultured renal cell lines have put forth strong evidence that hyperoxaluria is directly responsible for causing injury to renal epithelial cells, thereby putting into motion the process for the development of kidney stones [32]. *In vitro* studies have suggested that exposure of renal epithelial cells to oxalate and/or CaOx crystals causes an increase in expression of immediate early genes (c-*myc*, *Egr-1*, c-*jun* and *nur-77*) [33–35] and production of urinary macromolecules (Tamm-Horsfall protein, Osteopontin, Prothrombin fragment-1, Bikunin and inter-α-inhibitor, α_1_-Microglobulin, CD44, Calgranulin, Heparan sulfate, Osteonectin, Fibronectin, Matrix Gla Protein), modulating CaOx crystallization and crystal retention in the kidneys, as an adaptive responses of cells to oxalate [36]. Urinary Trefoil Factor 1 (TFF1) is an inhibitor of CaOx crystal growth, as it contains 4C-terminal glutamic residues that binds with calcium ions and prevents CaOx crystal growth [37]. Fibronectin, containing a tripeptide cell attachment sequence RGD, interacts with cells, thus inhibiting endocytosis of CaOx crystals, thereby protecting renal cells from oxalate induced injury [38]. Osteopontin, also contains this tripeptide sequence and a calcium binding site [39]. This protein has also shown to inhibit nucleation, aggregation, growth and cellular attachment of CaOx crystals [40,41]. Several other inhibitory urinary crystallization modulators are produced in kidneys and isolated from the kidney stones and urine [42].

Oxalate is a metabolite excreted by the kidney [43,44] that produces oxidant stress [32] and death of renal cells [45] at pathophysiological concentrations. High levels of oxalate exposure to renal cells may be responsible for varied morbidities seen in the kidneys, including CaOx nephrolithiasis [46,47]. For cell line based *in vitro* studies, the foremost step was to create a hyperoxaluric condition and this was achieved by exposing renal epithelial cells to 2mM sodium oxalate. The rationale behind this being that a salt of oxalate such as sodium oxalate, dissociates in liquid and in the presence of calcium ions (which are present in the body fluids or cell culture medium) forms insoluble calcium oxalate [48] which then can perpetuate the damage. According to the published research, addition of 1mM oxalate to cell-free media did not result in the formation of calcium oxalate crystals [28] and did not affect cell viability of renal cells [49]. Thus, this concentration was considered as the metastable limit [28] and it was further seen that in order to form CaOx crystals, the concentration of sodium oxalate required in DMEM [containing 1.8 mM Ca^2+^] needed to be greater than 0.5 mM [50]. Hence in our study, the concentration of oxalate ions and the duration for which the renal cells were exposed to these ions was in accordance to results of earlier reports and akin to environment inside the kidney as it is known that the urinary oxalate concentration changes as it traverses the nephron and is 0.22 mM in the excreted urine of non-stone formers, 0.44 mM in mild hyperoxaluria and 1.5 mM in conditions of primary hyperoxaluria [51]. Till date, several *in vitro* studies have been conducted in which the renal epithelial cells have been exposed to varying concentrations ranging from 0.1–4 mM oxalate ions [28,49,52,53].

In the present study, we identified and characterized four anionic proteins, namely, Nuclear pore anchor (A1), DEAD Box ATP-dependent RNA helicase 45 (B1), Lon protease homolog 1 (B2) and Heat shock protein 90–3 (C1), from the dried bark of *T*. *arjuna* which have the capability to inhibit CaOx crystallization and crystal growth and also have anti-apoptotic potential, thereby leading to cell survival. The anionic nature of the proteins is in line with documented research which states that proteins which are capable of inhibiting CaOx crystallization are anionic.

Nuclear pore anchor (NUA), is a 237 kDa protein and is conserved across plants and animals. NUA is localized to the inner surface of the nuclear envelope and is a component of the nuclear pore complex (NPC), which is large multiprotein complex consisting of more than 20 proteins and acts as a channel for exchange of macromolecules between the cytoplasm and the nucleus [54]. Around 67 percent of all the oxalate which is taken up by cells ends up in the nuclei of the cells of the liver and kidney and 40 percent of this is localized in the nuclear membrane. The presence of an oxalate binding protein in the NPC lends support to our study and that of others who have reported an increase in oxalate binding in experimental hyperoxaluria [55]. It has been further reported that increased expression of oxalate binding nuclear proteins leads to more than 50% increase in oxalate binding, seen in experimentally induced hyperoxaluria [56]. The presence of Nuclear pore anchor (NUA) in the *T*.*arjuna* extract having the capability of binding to the oxalate would thereby protect the renal cells from injury. Our results add proof to this theory and we have shown that this protein (A1), reduced the injury and apoptosis caused by oxalate to renal epithelial cells (NRK-52E and MDCK). The mechanism by which it could be leading to the attenuation of damage could be attributed to its composition. The NUA protein has a lysine rich region which could be involved in oxalate binding, thereby inhibiting the formation of COM crystals and reducing the injury to renal cells, by preventing interaction of oxalate to calcium and renal epithelial cells. In addition to lysine rich region, the presence of acidic polyglutamic acid residues may bind to calcium ions and thus, prevent the adhesion of COM crystals to the epithelial cell surface. Since COM crystals adhere to negatively charged cell surface molecules these could be inhibited by GAG, nephrocalcin, uropontin and various macromolecular components containing polyglutamic acid, polyaspartic acid, as well as citrate [14].

The second protein having antilithiatic potential purified from the *T*.*arjuna* extract showed sequence similarity to DEAD Box ATP-dependent RNA helicase 45. DEAD-box proteins are the largest and most characterized family of RNA helicases and contain a core of ~ 400 amino acids comprising seven to nine conserved motifs. The name is derived from its motif II that includes the sequence D-E-A-D (Asp-Glu-Ala-Asp) [57]. The cells are exposed to various stimuli which impose stress that can impact cell survival. Data suggests that DEAD/H RNA helicases have the ability to form stress granules, which promote cell survival by coordinating the stress signals [58]. These stress granules act as antioxidants by activating G3BP1 (GTPase-activating protein SH3 domain binding protein 1) and USP10 (ubiquitin-specific protease 10) in response to stress and protect cells from ROS-induced apoptosis [59]. The cell survival function of stress granules could be associated with suppression of ROS production during stress [59]. Since this protein is rich in glutamic acid and aspartic acid amino acids, this protein could bind calcium ions and thus, prevent the adhesion of COM crystals to the epithelial cell surface [14]. Our results have pointed to the ability of this protein (B1) which we had isolated and purified from *T*. *arjuna* extract, to protect the renal epithelial cells exposed to oxalate injury as evidenced by the various assays which showed an increase in cell viability and decreased levels of apoptotic cell death.

The third protein identified from the *T*. *arjuna* extract was Lon protease homolog 1, which is an ATP dependent mitochondrial protease [60] involved in the degradation of damaged and oxidized proteins of the mitochondrial matrix [61]. A common motif seen in various proteins which inhibit CaOx crystallization is the presence of the Arg-Gly-Asp (RGD) motif, examples being, nephrocalcin, THP, OPN, etc [62]. RGD cell attachment sequence was originally identified in fibronectin that binds to fibronectin receptor, integrin α_5_β_1_ [63]. It is known that the CaOx crystals bind to various anionic components present on the cell membrane. In addition, proteins such as nephrocalcin have the ability to bind the CaOx crystals at specific sites and thereby reduce their growth kinetics, as well as alter their morphology. Such a strategy could be followed by other proteins as well [14,64]. Therefore, it is very probable that the protein that we have identified, Lon protease homolog 1, may have a role similar to nephrocalcin. By binding via its RGD motif to the integrins present on the renal epithelial cells, this protein isolated from *T*.*arjuna*, could inhibit direct binding of CaOx crystals to cell surface and hence prevent the key step in stone formation i.e. crystal- cell interaction. Heparin, transforming growth factor-β2 (TGF-β2), and the tetrapeptide arginine-lycine-aspartic acid-serine (RGDS) negatively regulate the CaOx crystal endocytosis by interacting with cell adhesion sites on renal cell surface [38]. Studies by Lieske and colleagues have demonstrated diminution of crystal adhesion and internalization in BSC-1 cells upon treatment with RGDS, or fibronectin protein, containing this cell attachment sequence [38]. The degree of CaOx crystal deposition was inhibited by 60–80% in the cyclic RGD pretreated MDCK cells [65]. The presence of this protein could therefore act by inhibiting the binding of CaOx crystals to the renal cell surface and rescue cells from damage. In our present study we observed the cells treated with this protein indeed exhibited lower levels of apoptotic cell death owing to oxalate injury.

The fourth protein which we identified from the extract was, Heat shock protein 90–3, which is a molecular chaperone possessing antiapoptotic activity. It is a well-established fact that Heat-shock proteins (HSPs) are molecular chaperones which are induced by sub lethal cellular stresses, including temperature elevation, hypoxia and oxidative damage [66]. Hsp90 plays a key role in the refolding of denatured or damaged proteins and also in protein transport [67]. Hsp90 inhibits apoptosis and promotes cell survival by activating tumor necrosis factor-α which recruits receptor interacting protein (RIP) at the TNF receptor-1 to maintain NFκB activity [68]. Heat shock protein 90–3 also contains glutamic rich region which may aid in the binding of calcium ions and thus, prevent the adhesion of COM crystals to the epithelial cell surface [14]. In the light of these facts the attenuation of damage seen in the cells treated with this protein (B3) could be attributed to these functions.

The working hypothesis that was confirmed in this study is that, as per literature, oxalate and/or COM crystals induce oxidative stress that contributes to renal tubular epithelial cell injury, followed by death either by apoptosis and/or necrosis [69,70]. Most of the cells undergo apoptotic cell death and few cells die due to necrosis, and this may be in response to alterations in mitochondrial function that are characterized by initial increase in free-radical production, followed by a dissipation of the mitochondrial membrane potential and release of pro-apoptotic factors [71]. Oxalate damaged cells provide altered cell surface properties that act as a site for crystal adhesion [6] and cellular debris for crystal nucleation and growth [32]. In order to treat and prevent kidney stones, it is important to 1) target the interaction of free oxalate ions with the calcium ions and inhibit the formation of CaOx crystals and 2) avert the crystal retention to renal epithelial cells thus promoting cell survival.

## Conclusion

In this study we identified 4 novel proteins namely, Nuclear pore anchor, DEAD Box ATP-dependent RNA helicase 45, Lon protease homolog 1 and Heat shock protein 90–3, as anionic inhibitors of CaOx crystallization from the bark of *Terminalia arjuna*. We have put forth evidence that these anionic proteins possessed antilithiatic activity in terms of inhibition of CaOx crystallization and crystal growth kinetics. Further, these proteins protected the renal epithelial cells NRK-52E and MDCK from oxalate induced injury. A putative mechanism of action could be attributed to the anionic nature since, these proteins contain either polyglutamic acid, polyaspartic acid, polylysine rich regions and/or RGD sequence and these may be the factors which contribute to their antilithiatic activity. In addition, some of these proteins have anti-apoptotic activity and stimulate cell survival by inhibiting the pro-apoptotic factors. Our study points to the therapeutic value of the proteins present in *Terminalia arjuna* which may play a vital role in inhibiting the CaOx crystallization and open up exciting avenues to study therapeutic proteins from plants for the treatment of urolithiasis.

## Supporting Information

S1 Fig Flow Chart depicting procedure followed for obtaining the most potent proteins from *Terminalia arjuna* in terms of inhibition of CaOx crystallization. (TIF)
