# Supplementary figures and images for: Mechanistic Insights into the Antilithiatic Proteins from Terminalia arjuna: A Proteomic Approach in Urolithiasis
Source: PLoS One. 2016 Sep 20;11(9):e0162600. doi: 10.1371/journal.pone.0162600 (PMC5029924; doi:10.1371/journal.pone.0162600)

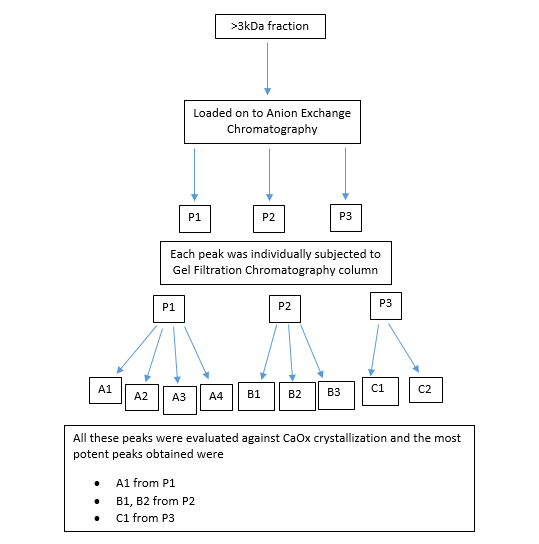

Supplement: S1 Fig — (TIF) [file pone.0162600.s001.tif]
